# Chemical control of electrical contact to *sp*^*2*^ carbon atoms

**DOI:** 10.1038/ncomms4659

**Published:** 2014-04-16

**Authors:** Thomas Frederiksen, Giuseppe Foti, Fabrice Scheurer, Virginie Speisser, Guillaume Schull

**Affiliations:** 1 Donostia International Physics Center (DIPC), Paseo Manuel de Lardizabal 4, E-20018 Donostia-San Sebastián, Spain; 2 IKERBASQUE, Basque Foundation for Science, E-48011 Bilbao, Spain; 3 Centro de Física de Materiales, Centro Mixto CSIC-UPV, Paseo Manuel de Lardizabal 5, E-20018 Donostia-San Sebastián, Spain; 4 Institut de Physique et Chimie des Matériaux de Strasbourg, UMR 7504 (CNRS–Université de Strasbourg), Strasbourg 67034, France

## Abstract

Carbon-based nanostructures are attracting tremendous interest as components in ultrafast electronics and optoelectronics. The electrical interfaces to these structures play a crucial role for the electron transport, but the lack of control at the atomic scale can hamper device functionality and integration into operating circuitry. Here we study a prototype carbon-based molecular junction consisting of a single C_60_ molecule and probe how the electric current through the junction depends on the chemical nature of the foremost electrode atom in contact with the molecule. We find that the efficiency of charge injection to a C_60_ molecule varies substantially for the considered metallic species, and demonstrate that the relative strength of the metal-C bond can be extracted from our transport measurements. Our study further suggests that a single-C_60_ junction is a basic model to explore the properties of electrical contacts to meso- and macroscopic *sp*^2^ carbon structures.

## Abstract (web summary)

Understanding metal-molecule contacts is crucial for molecular electronic devices. Here, the authors use a C60-terminated scanning tunnelling tip to probe how the chemical nature of the contacting atom on the substrate electrode determines the transport properties.

## Graphical abstract

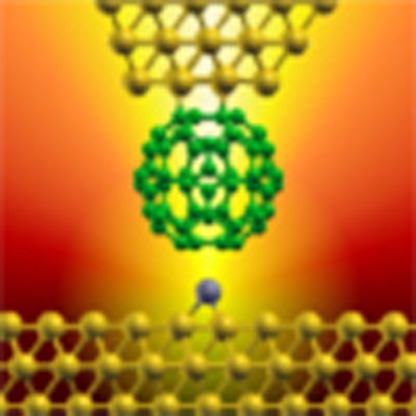

Thanks to their unique transport properties, carbon nanotubes (CNT), graphene sheets and nanoribbons are promising components for future nanoelectronics^1^. A close attention is paid to the connections of these graphitic structures to external metallic leads where the injection and the collection of charges are controlled^2^,^3^,^4^. Indeed, bad interfaces might ruin the transport properties of such carbon-based devices. To this respect, the chemical nature of the contacting leads is of major importance; it affects the electronic properties^5^ and, depending on the reactivity, the geometry of the contact^6^. The impact of these two aspects on the transport properties is entangled and, for mesoscopic structures, it is challenging to address them separately. Exploring the evolution of these parameters for contacts shrunk to the limit of individual atoms might solve this issue.

Scanning tunnelling microscopy (STM) contact experiments with fullerene molecules have revealed the decisive impact of atomic-scale modifications on transport properties^7^,^8^,^9^,^10^. Recently, some of us demonstrated that the conductance of a Cu–C_60_–Cu junction varies by more than an order of magnitude as a function of the number of Cu atoms in direct contact with the C_60_ molecule^11^. For small contact sections (that is, a single-atom contact) the conductance is limited by charge injection to the molecule, and depends essentially on the electronic and geometrical properties of the metal-C atomic contact, which acts as a conductance ‘bottleneck’.

Here we probe the charge injection efficiency at the interface between a C_60_ molecule and single metallic atoms of different chemical nature. We demonstrate that information on the reactivity between the fullerene and each specific atom can be extracted from our experiments, which match the properties observed at the mesoscopic scale. First-principles transport simulations with a novel scheme to describe the single-C_60_ junction reproduce the experimental findings and reveal how the chemical valence of the contacting atom determines the conductance of the junction^12^,^13^,^14^. Our study further demonstrates that atomic-scale junctions may be used to explore the properties of mesoscale graphitic contacts.

## Results

### Metal adatoms as chemically controlled electrodes

**Figure 1:**
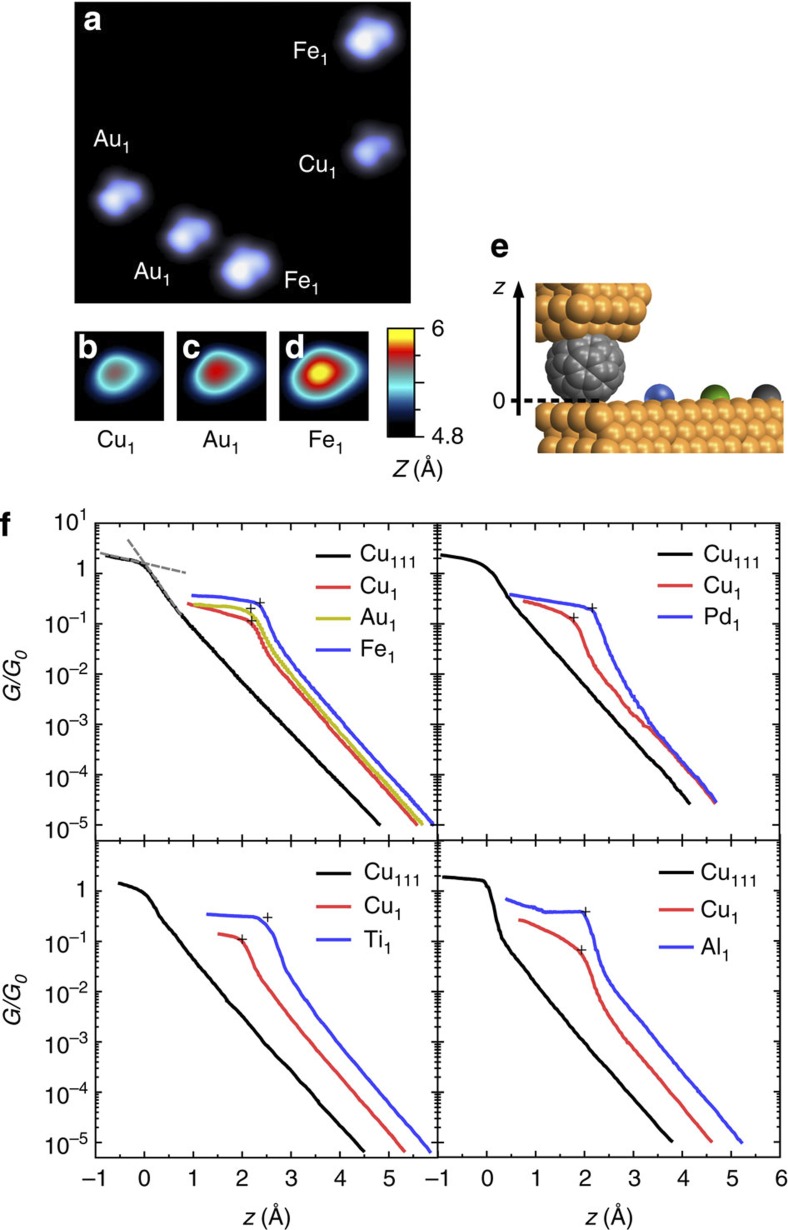
Individual metal adatoms contacted with a C_60_ -functionalized STM tip. (**a**) STM image (7.0 × 6.2 nm^2^) of different metal adatoms on Cu(111) acquired with a C_60_ tip at a sample voltage *V*=1.7 V. (**b**–**d**) Close-up views (1.4 × 1.4 nm^2^) of Cu_1_, Au_1_ and Fe_1_ images with a C_60_ tip for tunnelling conditions corresponding to the initial parameter of the traces in **f**. (**e**) Sketch of the C_60_ tip where *z*=0 corresponds to contact with the flat surface. (**f**) Experimental conductance traces *G*(*z*) in units of the conductance quantum *G*_0_=2*e*^2^/*h*. Black crosses mark the contact points defined as the intersection of the contact and transition regimes such as indicated by the dashed grey lines in panel **f** for the bare surface data.

To use different metal adatoms (M_1_=Cu_1_, Au_1_, Pd_1_, Fe_1_, Ti_1_, Al_1_) deposited on a Cu(111) surface as chemically controlled electrodes for molecular contacts, the apex of our STM tip was functionalized with a C_60_ molecule previously evaporated on the surface^15^. Figure 1a shows metallic adatoms imaged with a C_60_ tip at a sample voltage corresponding to the second lowest unoccupied molecular orbital (LUMO+1) of the molecular tip. The image reveals that this particular C_60_-functionalized tip is oriented with a bond between a pentagon and a hexagon (5:6 bond) towards the substrate^16^. Depending on the chemical nature of the adatom, a varying ‘apparent height’ is exhibited in the STM images as highlighted in the close-up views in Fig. 1b–d.

**Figure 2:**
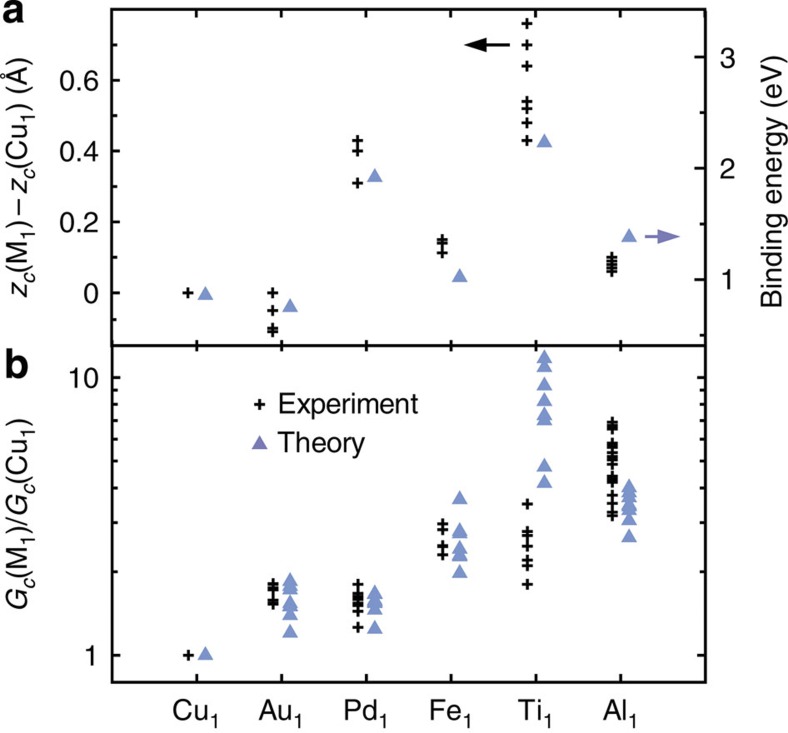
Mechanical and conductive trends with different adatom electrodes. (**a**) Experimental (black crosses) contact distances *z*_*c*_ and (**b**) contact conductances *G*_*c*_ for each of the considered metallic adatoms M_1_, obtained with different C_60_ tips. As a common reference, the data are (**a**) compared with or (**b**) normalized by the value obtained on Cu_1_. In panel **a**, the experimental contact distances are compared with calculated binding energies between an atom M_1_ and a single C_60_ molecule. In panel **b**, the experimental data are compared with calculated conductances (normalized by Cu_1_) for different electrode separations and binding sites (see text). The scattering in the theoretical data (blue triangles) corresponds to calculations for different lateral adatom positions and distances with respect to the C_60_ tip.

Figure 1e–f show contact experiments obtained with a C_60_-terminated STM tip and the different adatoms. As expected, the conductances vary exponentially with the tip approach in the tunnelling regime (large *z*). At shorter distances, a change in the slopes is observed, which marks a transition towards a contact regime. A contact distance *z*_*c*_ and a contact conductance *G*_*c*_ (black crosses in Fig. 1f) are determined for each conductance trace following the method detailed in ref. 11. We emphasize that the contact measurements performed on Au_1_, Cu_1_ and Fe_1_ were acquired with the same C_60_ tip and can be directly compared. The traces obtained on Pd_1_, Ti_1_ and Al_1_ pertain to other sets of measurements where they were compared with Cu_1_. Taking Cu_1_ as a reference, it is therefore possible to compare data from different experiments with different adatom species. Using this method, the impact of different molecular orientations and of different tip-side interfaces between C_60_ and metal on the experimental data is strongly reduced. Figure 2 and Table 1 summarizes the relative distances and conductances at contact.

### Mechanical aspects of contact formation

First we focus on the mechanical aspects of the different junctions. Figure 2a shows that the contact takes place at different tip-sample distances *z*_*c*_ depending on the chemical nature of the metallic adatom. For instance, the contact point occurs systematically at larger *z*_*c*_ values with Ti_1_ than with any of the other species. We find that the experimental variation in *z*_*c*_ correlates very well with binding energies *E*_*b*_ (blue triangles) between an atom M_1_ and a single C_60_ molecule calculated using density functional theory (DFT), see Methods section. The substrate has little impact on these binding energy trends, as discussed in Supplementary Fig. 1.

**Figure 3:**
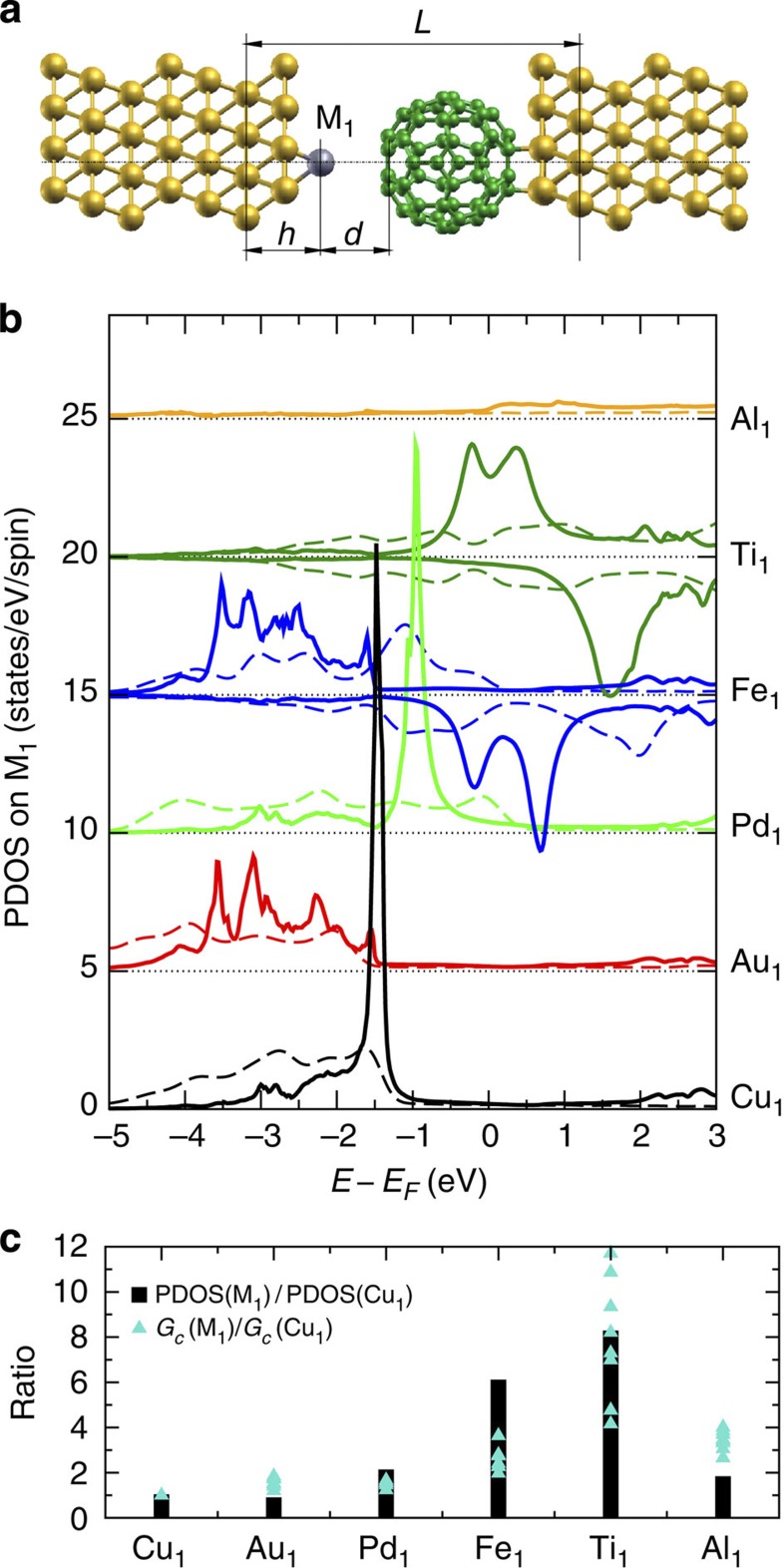
Analysis of calculated junction properties. (**a**) Model of the C_60_–M_1_ junctions considered in the calculations. (**b**) PDOS onto adatom basis (full lines: adatoms positioned in hollow sites on the molecular symmetry axis, *L*=18.5 Å) compared with the density of states for the same species in its bulk environment (dashed lines, Supplementary Fig. 5). For Fe and Ti, the two spin components are shown with opposite sign. The datasets are offset for clarity. (**c**) Comparison of adatom PDOS at the Fermi level (*L*=18.5 Å) with the calculated junction conductance *G*_*c*_(M_1_) at different electrode separations, normalized with respect to Cu_1_.

This suggests that the shape of the conductance trace around the point of contact is a measure of the attractive chemical forces between the adatom and the C_60_ tip. Indeed, when the C_60_ tip approaches the adatom, the attractive force between them increases giving rise to an elastic response^17^. The larger the attractive force between the molecule and the adatom, the ‘sooner’ a contact is established (that is, larger *z*_*c*_). Our DFT-generalized gradient approximation (GGA) calculations^18^,^19^ for the various junctions (Fig. 3a) also support that the variation observed in Fig. 2a is not due to the natural height variations of the different adatoms on Cu(111) (Supplementary Fig. 2). These findings also agree with measurements of the sticking behaviour of mesoscopic metallic electrodes on single-wall CNT^6^, supporting a hierarchy *E*_*b*_(Pd)>*E*_*b*_(Fe)>*E*_*b*_(Al)>*E*_*b*_(Au) for the binding energy. Theoretical works have revealed similar hierarchies for metallic adatoms^20^, clusters of metallic atoms^5^, and metal surfaces^21^ interacting with graphene sheets. While the reactivity between *sp*^2^ carbon atoms and metallic electrodes varies with the dimensionality (0D, 1D or 2D) and the curvature of the graphitic structure, it is noticeable that our atomic-scale experiment reproduces accurately all these trends.

### Chemical trends of the adatom on contact conductance

We next turn to the discussion of the contact conductance values. The experimental data in Fig. 2b (black crosses) reveal that *G*_*c*_ is on average about 1.6 times higher for Au_1_ and Pd_1_ than for Cu_1_ contacts. The largest average ratio was observed for Al_1_. Although we took the greatest care to locate the molecular tip over each adatom with the same relative position before the tip approach, sub-angström variations of this parameter are unavoidable. Such small variations can lead to different contact geometries and, consequently, to different contact conductances^9^. This explains the scattering in the experimental data in Fig. 2b. In our simulations we account for this aspect by considering different lateral positions of the adatoms with respect to the C_60_ tip^11^. We also consider different C_60_-adatom distances around the point of contact (Fig. 3a). The result of these simulations is displayed as blue triangles in Fig. 2b. An overall good agreement is obtained between experiment and theory. The conductance ratios are quantitatively reproduced for the Au_1_, Fe_1_ and Pd_1_ species. Ti_1_ and Al_1_ are also found to be the most conductive species, but contrary to the experiment theory assigns a larger conductance to Ti_1_ than to Al_1_. Our conductance ratios also agree with simulations for metal-graphene and metal-CNT interfaces^5^,^22^. Importantly, these results confirm that the efficiency of charge injection to a C_60_ molecule can vary substantially for different metal adatoms, independently of any geometrical considerations.

To rationalize the observed hierarchy, it is useful to consider the projected density of states (PDOS) on the different metallic adatoms as shown in Fig. 3b and Supplementary Fig. 3. For Cu_1_, Au_1_ and Pd_1_ a *d*-orbital adatom resonance is present significantly below the Fermi level *E*_*F*_. Around *E*_*F*,_ these three species as well as Al_1_ exhibit no particular spectral features corroborating that their *sp* electronic states of the free atoms hybridize strongly with Cu(111). The situation is very different for Fe_1_ and Ti_1_ which both exhibit a spin-polarized electronic structure with *d*-orbital resonances located around *E*_*F*_. For Fe_1_ (Ti_1_), the PDOS at *E*_*F*_ is dominated by the minority (majority) spin channel, rather analogous to the case of these metals adsorbed on graphene^20^. The transmission spin polarization^23^ is predicted to be as high as 88% in the case of Ti (Supplementary Fig. 4). Figure 3c compares the calculated junction conductances with the PDOS(*E*_*F*_) of the adatom at relatively large electrode separation (*L*=18.5 Å, Fig. 3a). To a first approximation, it is observed that the conductance is related to the PDOS.

This interpretation in terms of PDOS on the adatoms also offers an explanation for the overestimated conductance of Ti_1_ in the simulations. Close to resonant transport conditions—that is, when the adatom *d*-orbitals are nearly aligned with *E*_*F*_—the conductance depends sensitively on the resonance position and width. An inadequate description of the strong Coulomb repulsion between the 3*d* electrons localized on the adatom with standard DFT methods (and consequently their hybridization with the substrate) can therefore have a large impact on the calculated conductance. Although theory still provides a qualitative agreement with experiment in these cases (Fe_1_ and Ti_1_), a quantitative comparison needs to be taken with caution.

Figure 3b also compares PDOS of the various metal adatoms with the corresponding density of states in the bulk (Supplementary Fig. 5). Interestingly, despite of very different environments, their spectral features are rather similar. The main difference is obtained for Pd, where the *d*-band in bulk reaches *E*_*F*_ while it is located well below for Pd_1_ on Cu(111). This general correspondence supports the notion that our single adatoms can be considered representative for meso- and macroscopic electrodes. Therefore noble metals Cu_1_ and Au_1_ as well as Pd_1_ (closed *d*-shell elements) are generally less favourable for charge injection to C_60_ than the open *d*-shell elements Fe_1_ and Ti_1_ or the open *p*-shell element Al_1_. These observations suggest that the ability of a given metal to inject charges to *sp*^2^ carbon can, to some extent, be intuited from its chemical valence.

### Adatom influence on C_60_ orbitals

**Figure 4:**
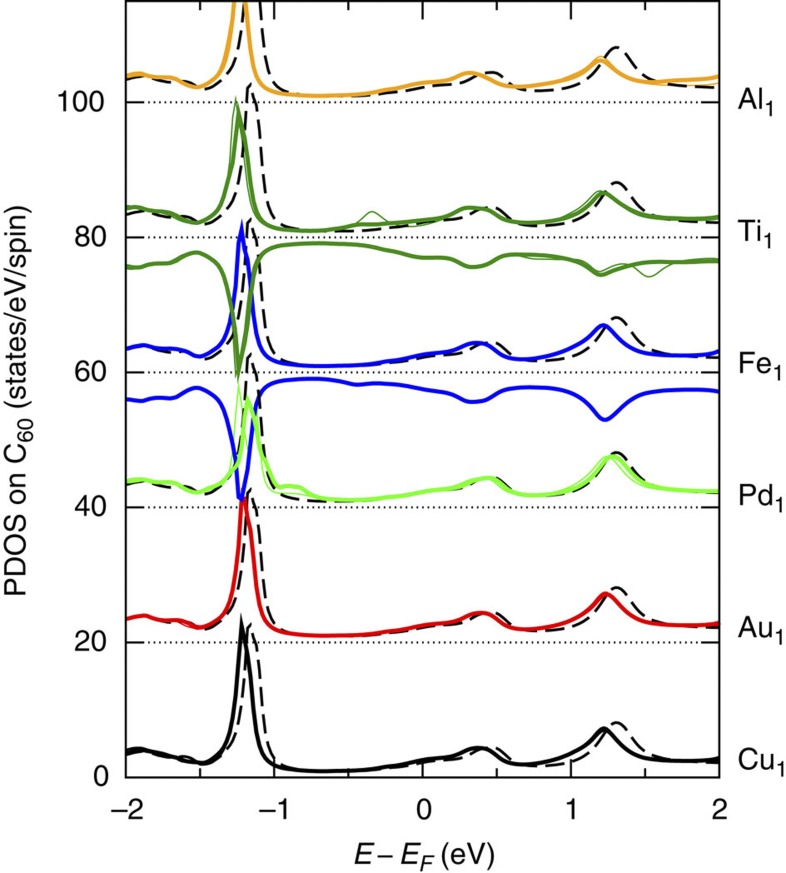
Influence of adatoms on the C_60_ orbitals. PDOS onto the C_60_ basis at relatively small electrode separation (*L*=17.2 Å. Fig. 3a). Adatoms in hollow sites on the molecular symmetry axis (thin lines) as well as adatoms shifted one hollow site away (thick lines) are considered. For Fe and Ti, the two spin components are shown with opposite sign. The datasets are offset for clarity and compared with PDOS for the isolated C_60_ tip (dashed black lines).

Finally, the electronic properties of the C_60_ are affected by the hybridization with the different adatoms^11^,^24^ with consequences for the junction conductance. Figure 4 shows the PDOS onto the C_60_ basis at relatively small electrode separation (*L*=17.2 Å, Fig. 3a). It reveals the characteristic spectral features of the C_60_ tip (essentially the highest occupied molecular orbital around −1.2 eV, the LUMO around 0.4 eV and the LUMO+1 around 1.2 eV), but also subtle differences depending on the adatom species. Compared with the PDOS for the isolated C_60_ tip (dashed black lines), it is observed that the spectrum is only weakly perturbed by the interaction with the adatoms. For Pd_1_, Fe_1_ and Ti_1,_ additional features appear coinciding with the *d*-resonances shown in Fig. 3b. The stronger impact of these adatoms on the C_60_ orbitals is consistent with their significant reactivities reported in Fig. 2a.

Figure 4 also reveals that the molecular resonances shift to lower energies as the contact is established, suggesting that these adatoms transfer additional electrons to C_60_ (for the full evolution with electrode separation *L*, see Supplementary Fig. 6). As a consequence, the LUMO becomes more resonant with the Fermi level and conductance is enhanced.^11^ These shifts are weak with Pd_1_ (little charge transfer) but strong with Al_1_ and Ti_1_ (significant charge transfer). The differences among the adatoms may therefore affect the conductance hierarchy for larger contacts (more than one atom); for example, the conductance would be expected to increase more rapidly with the number of atoms in the cluster for Al_1_ and Ti_1_ than for Pd_1_.

## Discussion

We have presented a systematic approach to probe the impact of electrode material on structure and electron transport at metal-*sp*^2^ carbon interfaces. DFT simulations confirm our observations and rationalize the observed properties. We find that some experimental parameters (the contact distance *z*_*c*_) directly reflect the strength of the metal-C bonds, but also that this strength does not necessarily reflect its conductive properties. In fact, we conclude that the ability of a metal atom to efficiently inject charges into a *sp*^2^ carbon atom is intimately linked to its chemical valence. For transition metals, it appears that elements in the centre of the *d*-block present a higher ability for charge injection than atoms from the *d*-block extrema due to a higher density of states at the Fermi level. Charge transfer between the metal adatom and the contacted C atoms of the C_60_ molecule modifies somewhat the position of the molecular resonances that also affects the conductance of the atomic-scale contact. Our single-C_60_ junctions are thus good models to explore the properties of electric contacts to *sp*^2^ carbon materials.

## Methods

### STM setup

The experiments were performed with an Omicron low-temperature STM operated at ≈4.5 K in ultrahigh vacuum (below 10^−10^ mbar). Cu(111) samples and etched W tips were prepared by Ar^+^ bombardment and annealing. W tips were indented into the sample surface to cover them with Cu. Approximately 0.2 monolayer of C_60_ molecules were deposited on a sample kept at room temperature. Au, Pd, Fe, Ti or Al atoms were deposited on the C_60_-covered Cu(111) surface kept at low temperature (4.5 K). Cu atoms were deposited using the method described in ref. 25.

### Identification of the different metallic species

**Figure 5:**
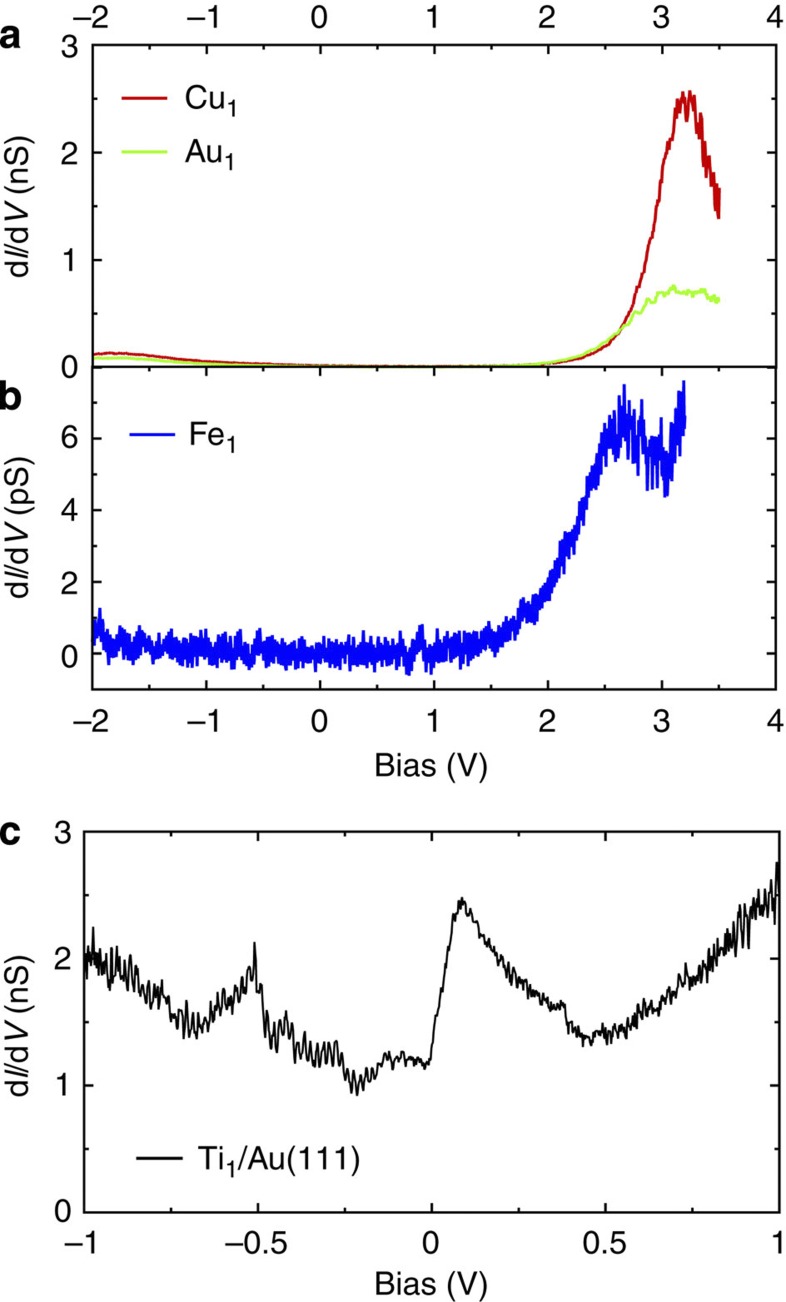
Experimental constant-height spectra of deposited adatoms recorded with a metallic STM tip. (**a**) Cu_1_, Au_1_ and (**b**) Fe_1_ deposited on Cu(111). Note that the spectrum in **b** was acquired with a lower current set point to limit instabilities at high voltages. These spectra are characteristic of the different species and are used to identify them. (**c**) Ti_1_ deposited on Au(111). The same features as those reported in ref. 27 confirm that we evaporated Ti_1_. The structure around ≈−0.5 V corresponds to the localization of the Au(111) surface state at the adatom.

In a first step, each of the metallic species (except Cu_1_) was evaporated individually (that is, different experimental sets) on a pristine Cu(111) sample maintained at low temperature. As referenced in the paper, Cu_1_ was systematically deposited by contacting the Cu(111) surface with the STM tip. For Cu_1_, Au_1_ and Fe_1_, it was possible to register constant height differential conductance (d*I*/d*V*) spectra over a large energy range (Fig. 5a,b). The d*I*/d*V* spectra were acquired using lock-in detection with a modulation frequency of 740 Hz and a root-mean-square modulation amplitude of 10 mV. These data reveal resonances characteristic of the different species. The d*I*/d*V* spectra of Cu_1_/Cu(111) are even referenced in the literature^26^. Using these signatures, it was easy to discriminate these three adatoms from each other on the surface.The d*I*/d*V* spectra of Pd_1_, Ti_1_ and Al_1_ could not be measured because of instabilities at high voltages. This is why these elements were measured separately and were only compared with Cu_1_ (which can be easily identified). It is therefore impossible to take one species for another.

**Figure 6:**
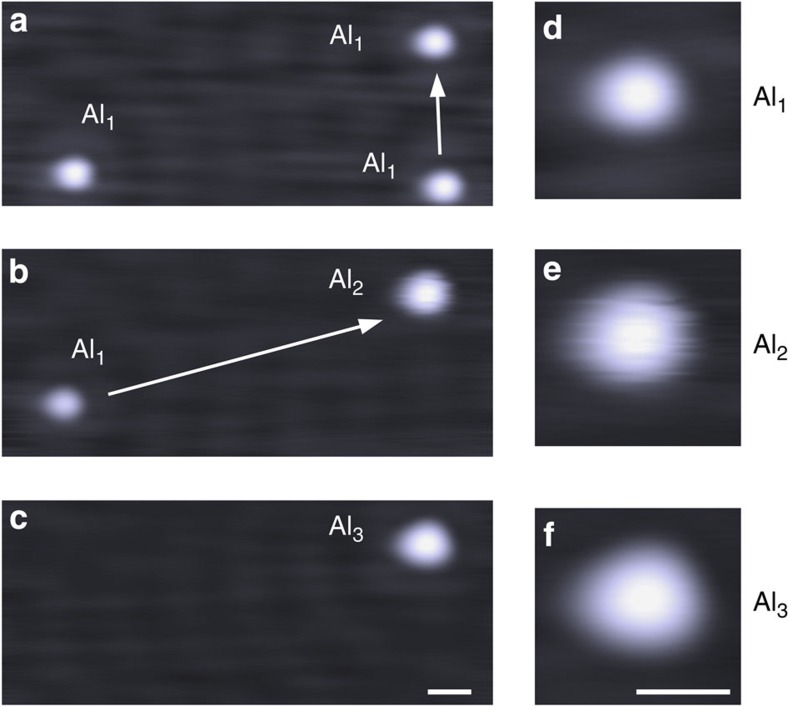
Experimental STM images of a manipulation sequence on Cu(111). (**a**) Three individual Al_1_ are arranged into (**b**) Al_1_ and a dimer Al_2_, and finally into (**c**) a trimer Al_3_. Panels (**e**,**f**) show image close ups of the cluster to the upper right. The scale bar, 1 nm.

We had a particularly critical look at the Ti_1_ and Al_1_ cases. Although the evaporation of Cu_1_, Au_1_, Pd_1_ and Fe_1_ is well known and relatively easy to control, Ti_1_ and Al_1_ are less documented. Ti_1_ was deposited by T. Jamneala *et al*.^27^ on Au(111). In this case, d*I*/d*V* data reveal a small Kondo feature at the Fermi level as well as a characteristic feature around 150 mV (most likely a 3*d*-orbital resonance). We were able to reproduce these features (Fig. 5c), which validates the evaporation of Ti_1_. Unfortunately, Al_1_ reveals no features in d*I*/d*V* spectroscopy and this strategy can thus not be used for the identification. To investigate if the evaporated material really corresponds to individual atoms (and not clusters of atoms), we used atom manipulation techniques to form dimers Al_2_ and trimers Al_3_ (Fig. 6). The fuzzy image in Fig. 6e is a strong indication of a dimer, similar to the Cu_2_/Cu(111) case^26^. While we cannot rule out a possible contamination of the deposited adatoms, we believe the manipulation sequence is a strong support for Al_1_.

### C_60_–M_1_ binding energy calculations

The binding energy *E*_*b*_ between a C_60_ molecule and a single metallic atom M_1_ is defined as *E*_*b*_(C_60_M_1_)=*E*_tot_(C_60_)+*E*_tot_(M_1_)−*E*_tot_(C_60_M_1_), where *E*_tot_(*i*) is the total energy of system *i* from a spin-polarized calculation. A positive binding energy thus corresponds to a stable system. DFT calculations were carried out with the Vasp code^28^ using a 515-eV planewave cutoff, the generalized gradient approximation (GGA) of Perdew-Burke-Ernzerhof (PBE) for the exchange-correlation functional^29^, a tetragonal supercell of 14 × 14 × 20 Å^3^, a Gaussian smearing of 1 meV for the occupancies, and relaxations until residual forces were smaller than 0.02 eV Å^−1^. Constraints were imposed to fix the metal atom to different binding sites on the C_60_ cage. The results for the binding energies *E*_*b*_, spin magnetic moment *μ* and characteristic bond lengths are given in Tables 2 and 3. These trends are mostly in agreement with those reported in ref. 23. We checked the role of applying a dipole correction for electrostatic interactions between neighbouring cells along the dimer axis, but this only affects the binding energies by a few percent for our simulation cells (Supplementary Table 1).

### Electronic structure of model junctions

The electronic structure for the various junction geometries (Fig. 3a) was calculated with the Siesta^18^ pseudopotential DFT method with the GGA-PBE exchange-correlation functional^29^ as described in refs 11 and 15. The junctions are modelled by structures comprizing a 13-layer slab Cu(111) in a 4 × 4 representation, the adatom as well as the C_60_ molecule. A standard single-zeta plus polarization basis was employed for bulk Cu (0.15-eV energy shift) and a long-ranged (0.02-eV energy shift), double-zeta plus polarization basis for C_60_ and the adatoms. The pseudopotentials were constructed according to the parameters specified in Supplementary Table 2. Real-space grid integrations were carried out using a 200 Ry energy cutoff. The 3D Brillouin zone was sampled with a 2 × 2 × 1 Monkhorst-Pack *k*-mesh. The lattice constant for the Cu crystal was set to 3.70 Å. As a function of varying the electrode separation *L* (Fig. 3a), the adatoms and underlying surface layer were relaxed to 0.02 eV Å^−1^. The adatom heights *h* and radial distances *r* to the molecular symmetry axis are reported in Supplementary Fig. 7. PDOS were calculated using Inelastica^30^ with a 13 × 13 *k*_||_-mesh of Gauss–Kronrod points and a broadening of *η*=0.1 eV in the bulk part of the semi-infinite electrodes.

### First-principles transport simulations

The electronic structure from Siesta was used to calculate the transport properties for the TranSiesta^19^ setup using Inelastica^30^. The zero-bias conductance *G* is determined by the electron transmission function *T*(*E*) evaluated at the Fermi energy *E*_*F*_:


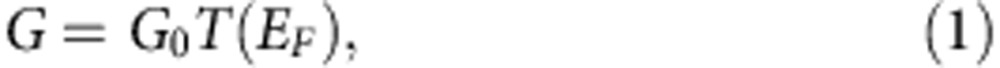


where *G*_0_=2*e*^2^/*h* is the conductance quantum. We calculate the electron transmission in two different approaches as described below.

The typical approach is to calculate *T*(*E*_*F*_) per unit cell for a system which is periodically repeated in the plane perpendicular to the electron transport. This periodic (P) approach is consistent with the DFT treatment that relies on this periodicity. As a consequence of Bloch’s theorem, the electron transmission is averaged over *k*-points in the 1st Brillouin zone (with weights *w*_*k*_=1/*N*_*k*_ when using a linearly spaced mesh with *N*_*k*_ points):


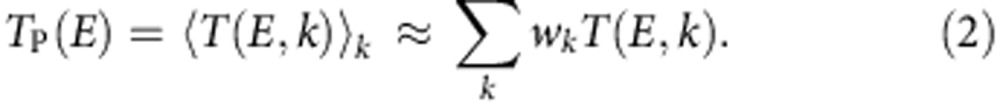


Here *T*(*E*,*k*) is the electron transmission resolved in terms of electron momentum *k*


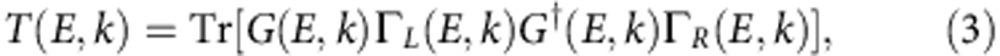


and where the retarded Green’s function *G* in the device region is


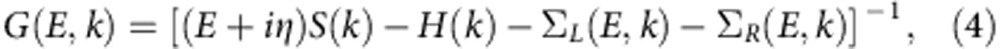


with *S*(*k*) and *H*(*k*) being the overlap and Hamiltonian matrices, respectively. The electrode-coupling rates are related to the self-energies via


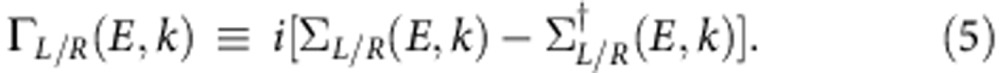


The experiment, however, concerns a single C_60_-functionalized STM tip in contact with a single adatom on Cu(111). Our alternative computational approach, more in line with this situation, corresponds to partitioning the system into periodic leads (where quantities are *k*-sampled) and a ‘non-periodic’ device region (which does not depend on *k*). This is similar to a real-space DFT approach for STM simulations^31^,^32^ and methods used for phonon transport through nanoconstrictions^33^,^34^. For our simulation cells, the natural choice is to consider the adatom and C_60_ as the device, since the electronic coupling between these atoms and their periodic repetitions is significantly smaller than for the Cu atoms in the two semi-infinite electrodes. The transmission *T*_NP_(*E*) is then given by


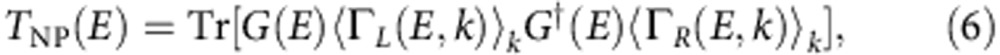


where now the device Green’s function *G* is thought to concern a single junction


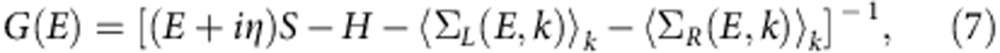


with only the self-energies being sampled over *k*. In practice, since the device region may not be completely decoupled from its periodic repetitions, we sample *T*_NP_(*E*) over a coarse 3 × 3-*k*-mesh for the device part (*H* and *S*) and check that there are only small variations on this mesh.

**Figure 7:**
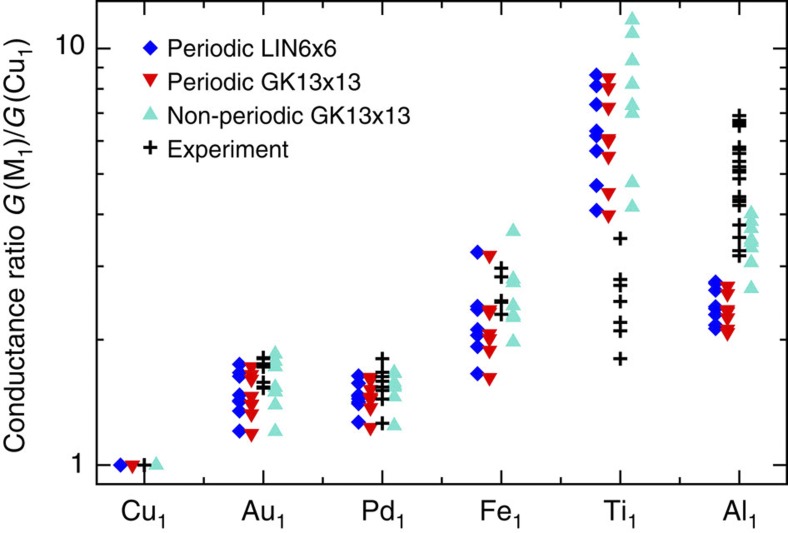
Comparison of conductance ratios with different computational approaches. Calculations for periodic arrays of C_60_ junctions with the transmission *T*(*E*_*F*_) sampled on a *k*-mesh of 6 × 6 linearly spaced points (blue diamonds) or with 13 × 13 Gauss–Kronrod points (red triangles) in 1st Brillouin zone (1BZ). Calculations for a ‘non-periodic’ molecular junction with electrode self-energies Σ_*L*/*R*_ sampled on a *k*-mesh of 13 × 13 Gauss–Kronrod points in 1BZ (turquoise triangles). The device region consists of the adatom and C_60_. The experimental ratios (black crosses) are shown for comparison.

Figure 7 compares the conductance ratios for the two computational schemes as well as for different sampling of *k*-space (6 × 6 linearly spaced points versus 13 × 13 Gauss–Kronrod points). We find that the latter ‘non-periodic’ approach, implemented on this occasion in Inelastica^30^, gives a slightly better overall agreement with the experimental conductance ratios (in particular for the Al_1_ case). The figure also highlights the convergence in *k*-space as essentially the same conductance ratios are obtained for the two periodic sets of calculations (blue diamonds versus red triangles). In the Gauss–Kronrod scheme, a broadening of *η*=0.1 eV was used in the bulk electrode.

The absolute conductances for both schemes are shown in Supplementary Figs 8 and 9. Further, for the spin-polarized species (Fe and Ti) the transmission per spin channel as well as the transmission spin polarization is shown in Supplementary Fig. 4. We also checked that the calculated conductance ratios for Al_1_ shows no significant dependence on the C_60_-orientation (Supplementary Fig. 10).

### Kondo physics

We note that eventual Kondo physics^35^,^36^, beyond the DFT methods employed here, is not expected to impact our results. No Kondo resonances were experimentally resolved in d*I*/d*V* for any of the considered species on Cu(111). Therefore, even if a narrow Kondo feature would exists for species like Fe_1_ and Ti_1_, it has a negligible effect on the measured conductance at *V*=−50 mV.

## Author contributions

The experiment was conceived by G.S. and carried out by G.S., F.S. and V.S. T.F. and G.F. performed the simulations. T.F. and G.S. wrote the paper with comments and input from all authors.

## Additional information

**How to cite this article:** Frederiksen, T. *et al*. Chemical control of electrical contact to sp2 carbon atoms. *Nat. Commun.* 5:3659 doi: 10.1038/ncomms4659 (2014).

## Supplementary Material

Supplementary Information Supplementary Figures 1-10 and Supplementary Tables 1-2

## Figures and Tables

**Table 1:** Absolute experimental contact distances 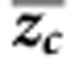 and contact conductances 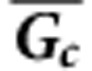.

**Adatom**	**Cu_1_**	**Au_1_**	**Pd_1_**	**Fe_1_**	**Ti_1_**	**Al_1_**
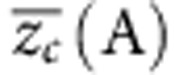	1.9±0.2	1.9±0.2	2.3±0.2	2.1±0.2	2.5±0.3	2.0±0.2
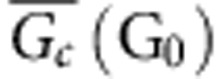	0.11±0.02	0.18±0.05	0.17±0.05	0.29±0.08	0.28±0.10	0.65±0.20

The estimates for each adatom species are determined according to these expressions: 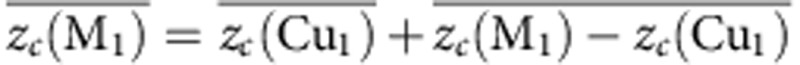 and 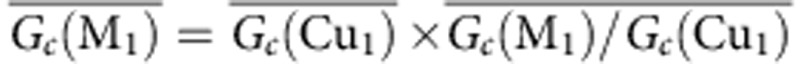. The absolutes values for Cu_1_ were deduced from the overall experimental data sets.

**Table 2:** Calculated binding energies and spin magnetic moments of C60-M_1_ clusters.

	**Al**_**1**_	**Au**_**1**_	**Co**_**1**_	**Cu**_**1**_	**Fe**_**1**_	**Pd**_**1**_	**Ti**_**1**_
5:6 bond	1.23 (1)	0.58 (1)	1.32 (1)	0.87 (1)	1.02 (4)	1.69 (0)	1.61 (4)
6:6 bond	1.38 (1)	0.55 (1)	1.67 (1)	0.74 (1)	1.03 (2)	1.93 (0)	2.06 (2)
Corner	1.26 (1)	0.75 (1)	1.15 (3)	0.87 (1)	0.92 (4)	1.56 (0)	1.45 (4)
Hexagon	1.22 (1)	—	1.14 (1)	0.18 (1)	1.03 (2)	0.95 (0)	2.30 (2)
Pentagon	1.39 (1)	—	1.23 (1)	0.42 (1)	0.61 (4)	1.26 (0)	1.84 (4)

Vasp GGA-PBE binding energies *E*_*b*_ in eV (spin magnetic moment μ in μ_*B*_) for different metallic species M_1_ bonded at specific sites of a C_60_ molecule. Non-bonding configurations are indicated with —.

**Table 3:** Calculated bond lengths of C60-M_1_ clusters.

	**Al**_**1**_	**Au**_**1**_	**Co**_**1**_	**Cu**_**1**_	**Fe**_**1**_	**Pd**_**1**_	**Ti**_**1**_
5:6 bond	2.30	2.29	1.94	2.07	2.07	2.12	2.25
6:6 bond	2.32	2.29	1.92	2.06	1.98	2.09	2.09
Corner	2.18	2.12	1.93	1.95	1.98	2.01	2.19
Hexagon	2.66	—	2.17	2.46	2.18	2.50	2.28
Pentagon	2.53	—	2.10	2.37	2.35	2.41	2.35

Vasp GGA-PBE results for the average bond length (in Å) between the metallic species M_1_ and the bonding-C atoms.Non-bonding configurations are indicated with —.
