# Supplementary material for: Chemical control of electrical contact to sp2 carbon atoms
Source: Nat Commun. 2014 Apr 16;5:3659. doi: 10.1038/ncomms4659 (PMC3997807; doi:10.1038/ncomms4659)
Supplement: Supplementary Information — Supplementary Figures 1-10 and Supplementary Tables 1-2 [file ncomms4659-s1.pdf]

# Supplementary Information:
# Chemical control of electrical contact to $sp^2$ carbon atoms

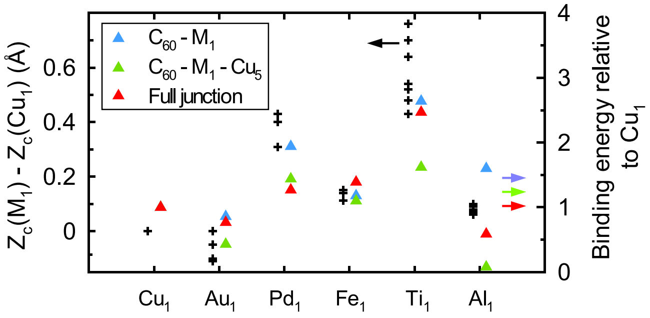


Supplementary Fig. 1. **Comparison of experimental contact distances and calculated binding energies.** Experimental contact distances $z_c(\mathrm{M}_1)$ (black crosses) for each of the considered metallic adatoms $\mathrm{M}_1$ (obtained with different $\mathrm{C}_{60}$-tips) compared with relative VASP GGA-PBE binding energy trends for three different systems (i) an isolated $\mathrm{C}_{60}$ binding to a single isolated atom $\mathrm{M}_1$ (cf. Tab. II of the main text, blue triangles), (ii) an isolated $\mathrm{C}_{60}$ binding to a metal cluster $\mathrm{M}_1\mathrm{Cu}_5$ ($\mathrm{C}_{60}$ contacted by $\mathrm{M}_1$, green triangles), and (iii) VASP energy gain for the SIESTA junction geometries [see Fig. 3(a)] by reducing the electrode separation from $L = 18.5$ Å to $L = 17.2$ Å (red triangles). The correlation of experimental and theoretical data suggests that the experimental contact distance $z_c$ is mainly determined by the binding energy strength to the metal adatom.

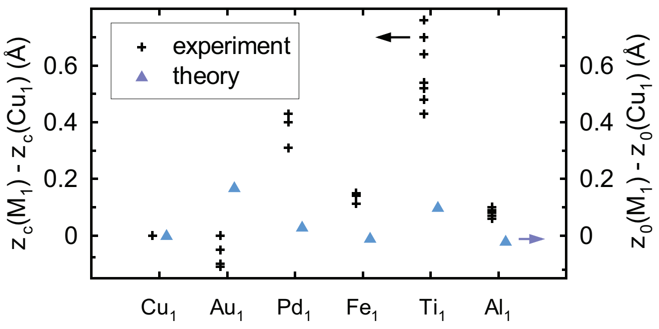


Supplementary Fig. 2. **Comparison of experimental contact distances and calculated adatom heights.** Experimental contact distances $z_c(\mathrm{M}_1)$ (black crosses) for each of the considered metallic adatoms $\mathrm{M}_1$ (obtained with different $C_{60}$-tips) compared with the calculated initial heights (blue triangles) of the atom $\mathrm{M}_1$ on Cu(111). As a common reference the data are compared to the value obtained on $Cu_1$.

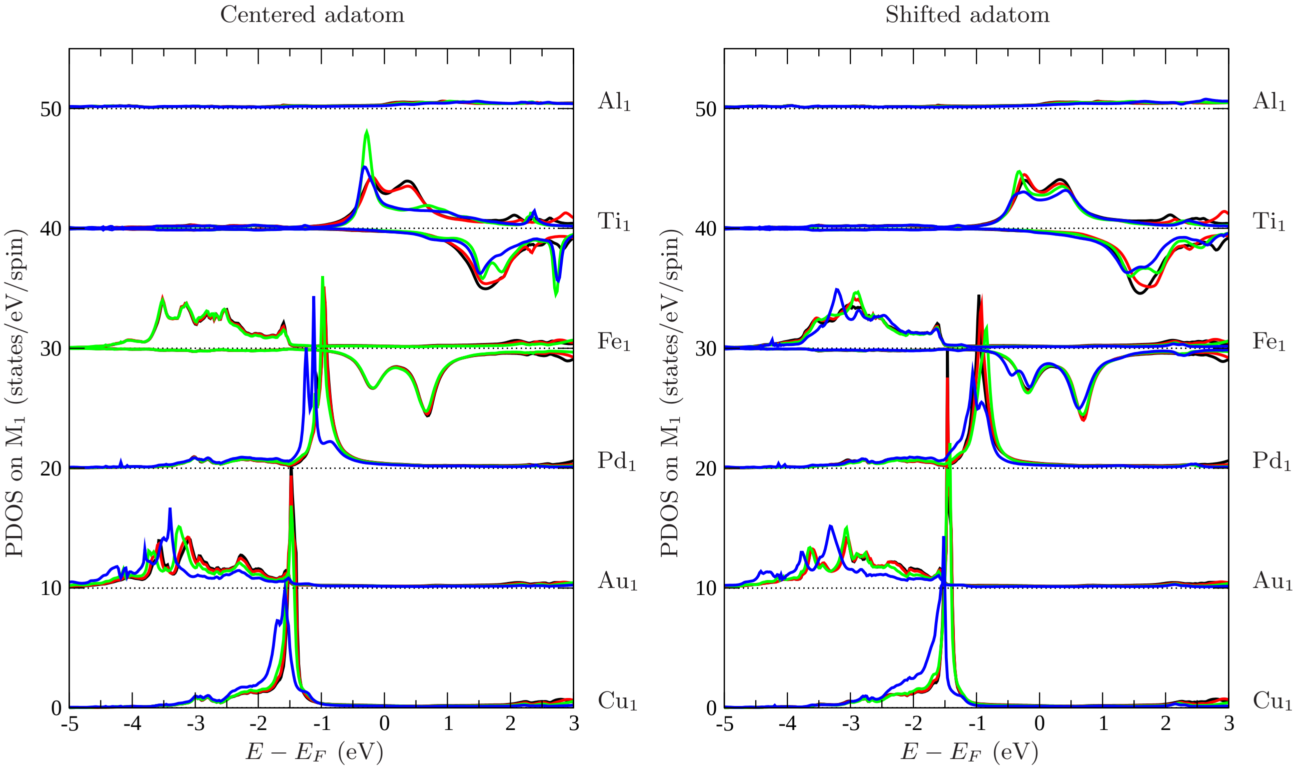


Supplementary Fig. 3. **Projected density of states (PDOS) onto adatom basis orbitals.** The left (right) plot corresponds to geometries where the adatom is aligned on (shifted one hollow-site with respect to) the molecular symmetry axis. The electrode separations are $L = 18.5$ Å (black), $L = 18.0$ Å (red), $L = 17.6$ Å (green), and $L = 17.2$ Å (blue). The datasets are offset for clarity. Note that we obtained no stable "centered" $Fe_1$ geometry at $L = 17.2$ Å (it relaxes into the shifted geometry). A Gauss-Kronrod $k$-mesh of $13 \times 13$ was used in combination with a smearing of $\eta = 0.1$ eV in the semi-infinite electrodes.

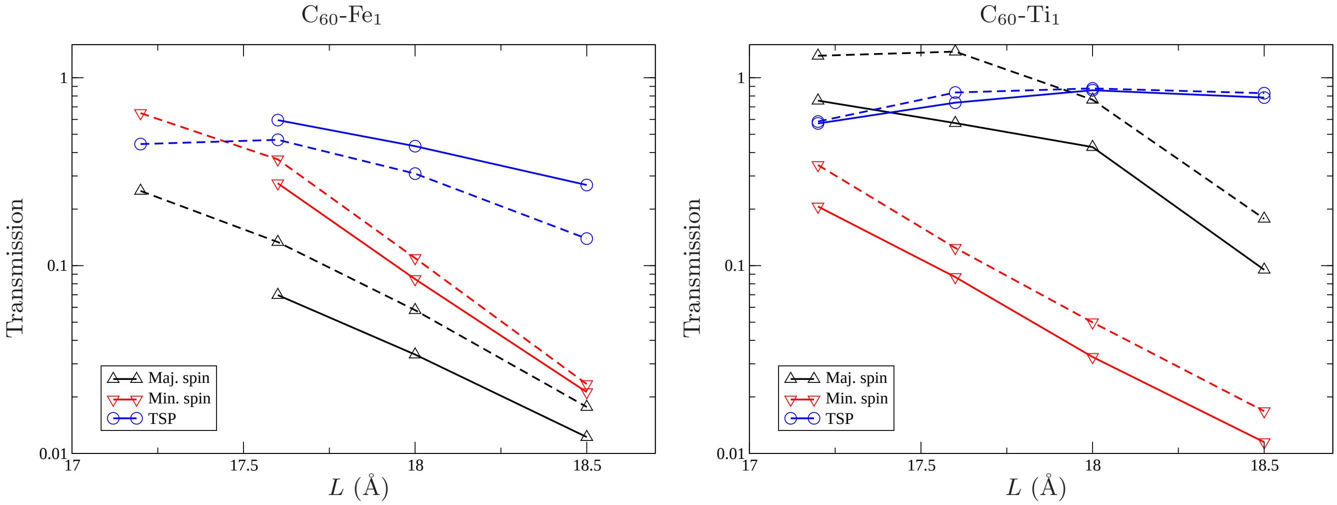


Supplementary Fig. 4. **Total transmission $T_{\mathrm{NP}}(E_F)$ vs electrode separation $L$ for majority and minority spin channels in the "non-periodic" treatment.** The transmission spin polarization (TSP) is defined as $|T_{\mathrm{maj}} - T_{\mathrm{min}}|/(T_{\mathrm{maj}} + T_{\mathrm{min}})$. The full (dashed) lines correspond to geometries where the adatom is aligned on (shifted one hollow-site with respect to) the molecular symmetry axis. The electrode self-energies $\Sigma_{L/R}$ are sampled using a $k$-mesh with $13 \times 13$ Gauss-Kronrod points in 1BZ (broadening of $\eta = 0.1$ eV in the bulk electrode). The non-periodic device region consists of the adatom and $C_{60}$.

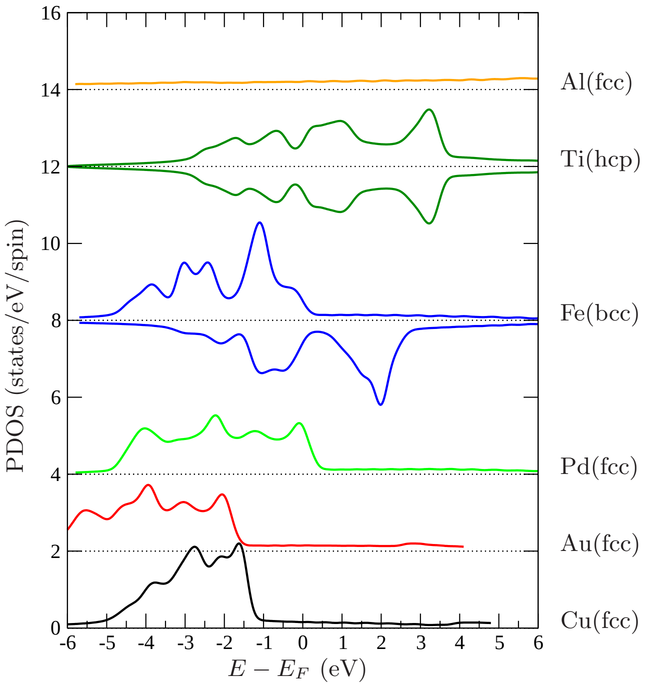


Supplementary Fig. 5. **Density of states per atom in the bulk materials.** Calculations were performed with SIESTA using a $k$-mesh of $40 \times 40 \times 40$ and smearing $\eta = 0.2$ eV. Data is offset for clarity.

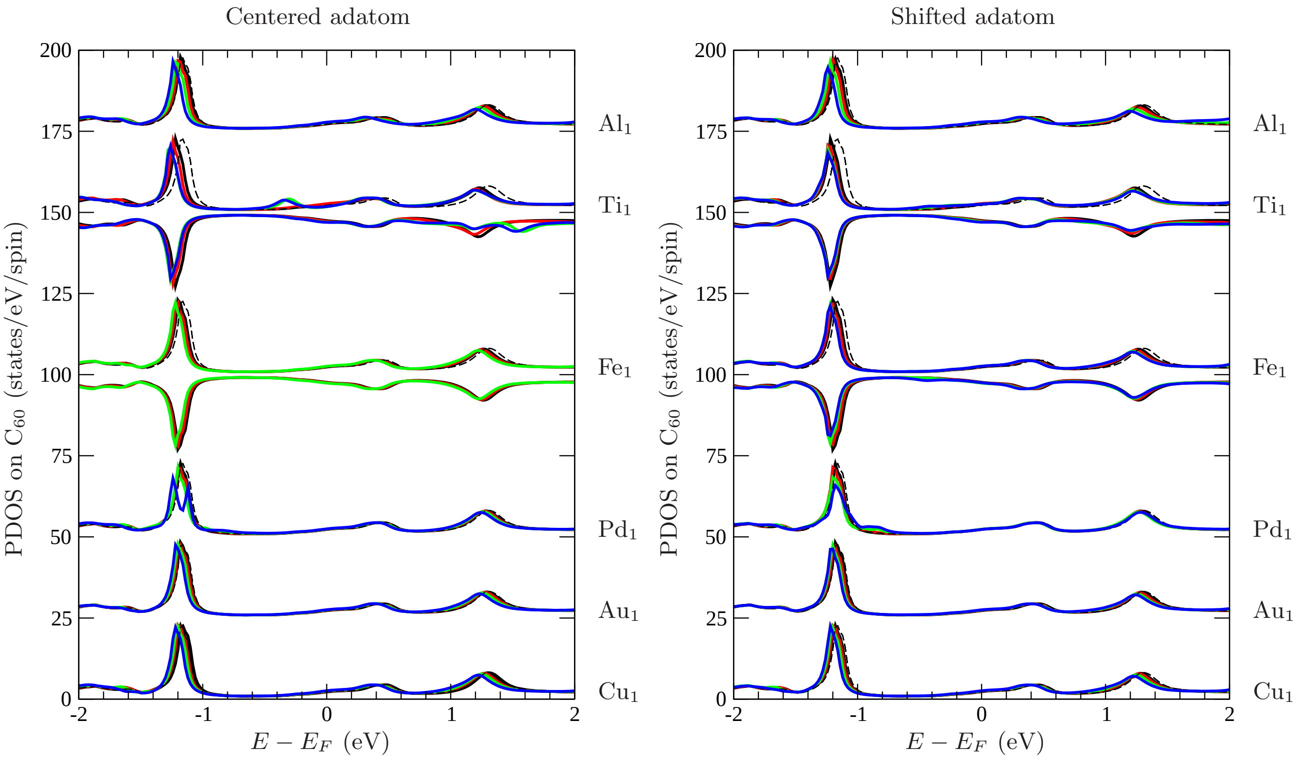


Supplementary Fig. 6. **Projected density of states (PDOS) onto the $C_{60}$ basis orbitals.** The left (right) plot corresponds to geometries where the adatom is aligned on (shifted one hollow-site with respect to) the molecular symmetry axis. The electrode separations are $L = 18.5$ Å (black), $L = 18.0$ Å (red), $L = 17.6$ Å (green), and $L = 17.2$ Å (blue). The datasets are offset for clarity and compared with PDOS for the isolated $C_{60}$-tip (dashed black lines). Note that we obtained no stable "centered" $Fe_1$ geometry at $L = 17.2$ Å (it relaxes into the shifted geometry). A Gauss-Kronrod $k$-mesh of $13 \times 13$ was used in combination with a smearing of $\eta = 0.1$ eV in the semi-infinite electrodes.

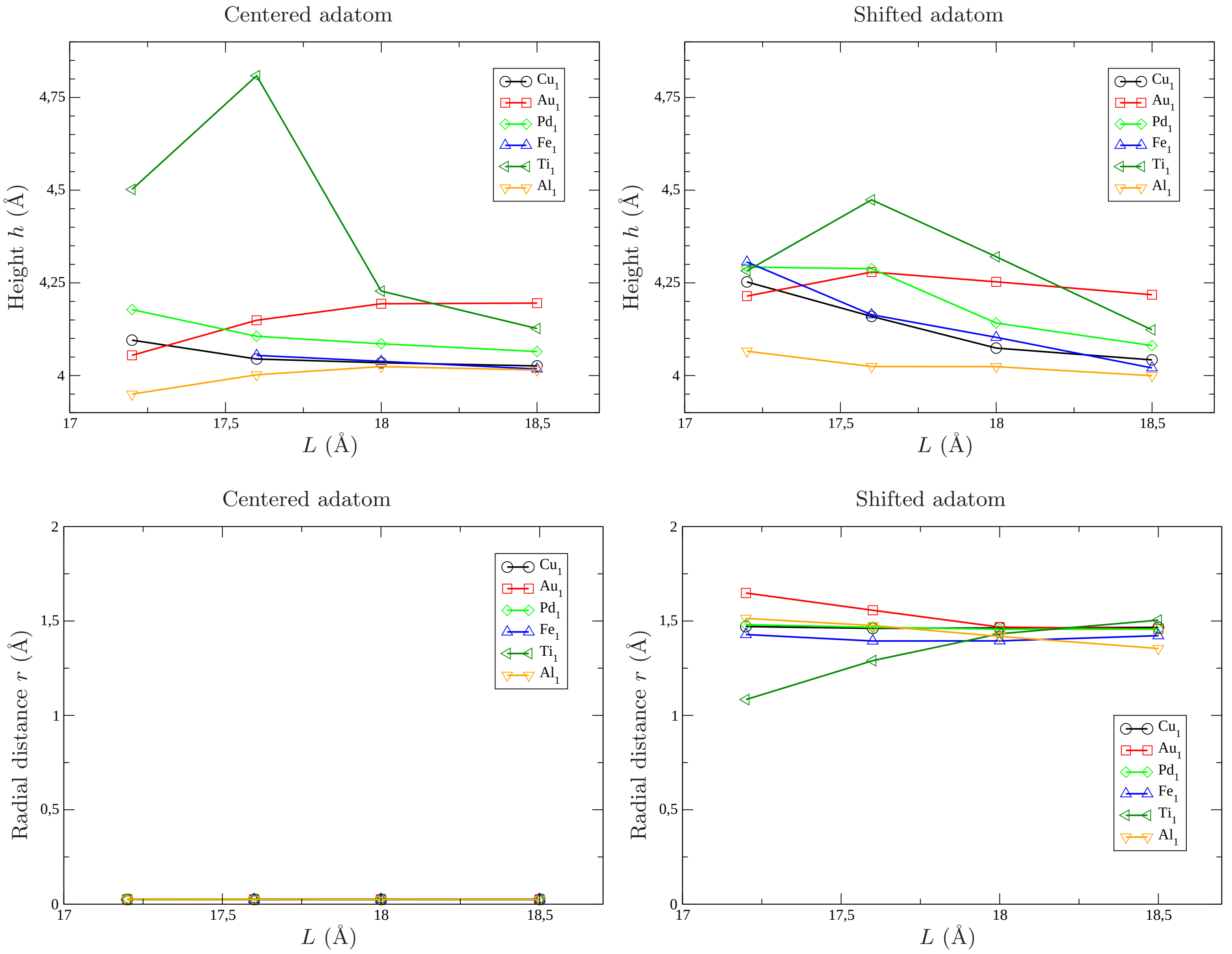


Supplementary Fig. 7. **Geometric properties of the simulated junctions.** (top) Adatom height $h$ and (bottom) radial distance $r$ from molecular symmetry axis vs electrode separation $L$.

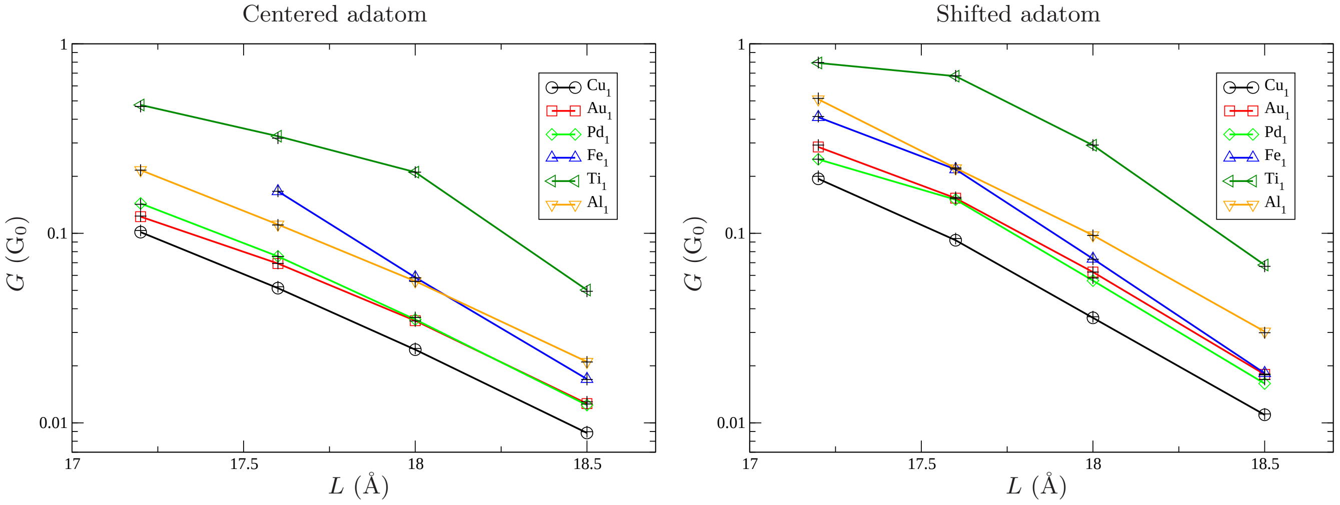


Supplementary Fig. 8. **Zero-bias conductance $G = \mathrm{G}_0 T_\mathrm{P}(E_F)$ vs electrode separation $L$ calculated for a periodic array of junctions.** The transmission $T(E_F)$ is sampled over a $k$-mesh with either $6 \times 6$ linearly spaced points [colored symbols] or with $13 \times 13$ Gauss-Kronrod points (broadening of $\eta = 0.1$ eV in the bulk electrode) [black crosses].

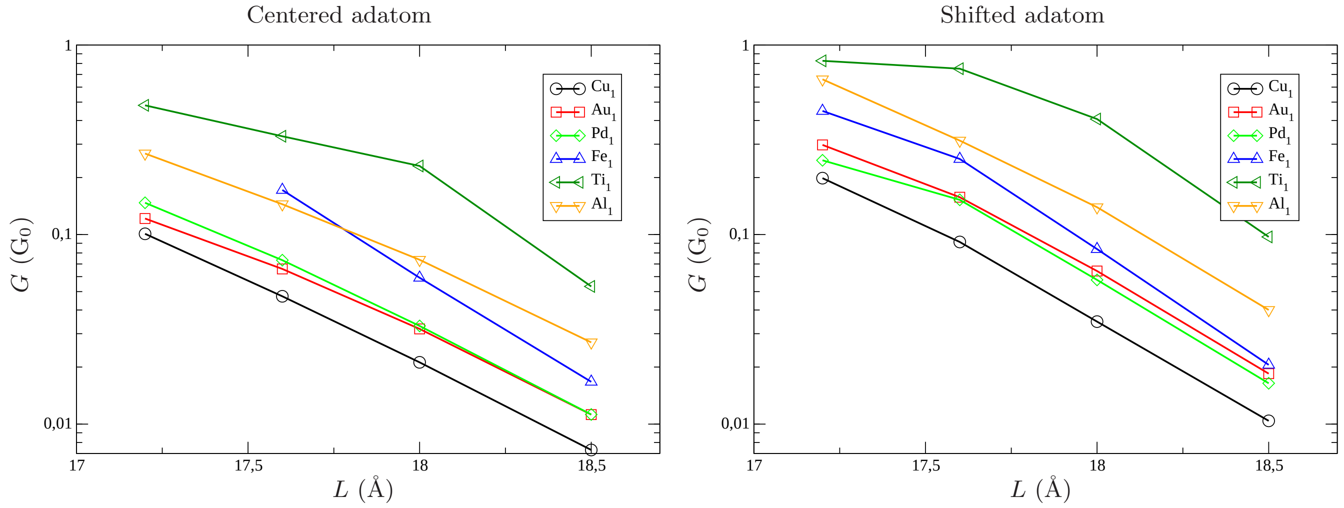


Supplementary Fig. 9. **Zero-bias conductance $G = G_0 T_{\mathrm{NP}}(E_F)$ vs electrode separation $L$ calculated for a single molecular junction with the "non-periodic" scheme.** The electrode self-energies $\Sigma_{L/R}$ are sampled using a $k$-mesh with $13 \times 13$ Gauss-Kronrod points in 1BZ (broadening of $\eta = 0.1$ eV in the bulk electrode). The non-periodic device region consists of the adatom and $C_{60}$.

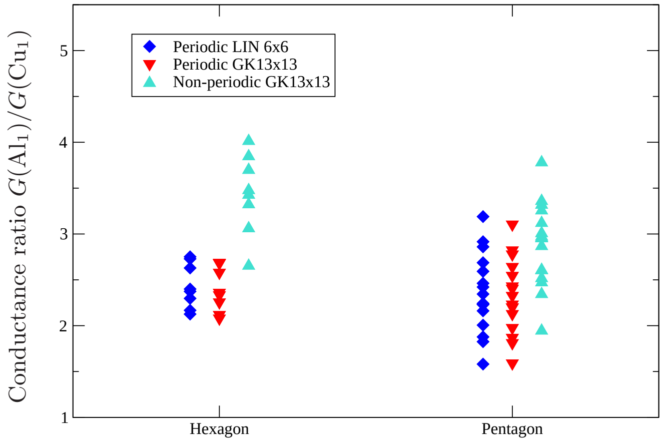


Supplementary Fig. 10. **Effect of $C_{60}$ orientation on the conductance ratios $G_c(\mathbf{Al}_1)/G_c(\mathbf{Cu}_1)$.** Different adatom positions on the surface (on the molecular symmetry axis or shifted a hollow-site away) and different $C_{60}$-adatom distances around the point of contact are considered. This shows that the orientation of the $C_{60}$ tip is not playing an essential role for the conductance ratios (but the absolute conductance values are significantly higher with a pentagon orientation than with a hexagon). The same three computational approaches as discussed in Fig. 6 of the main text were applied.

| | $Al_1$ | $Au_1$ | $Co_1$ | $Cu_1$ | $Fe_1$ | $Pd_1$ | $Ti_1$ |
|---|---|---|---|---|---|---|---|
| 5:6 bond | 1.22 (1) | 0.58 (1) | 1.33 (1) | 0.86 (1) | 1.02 (4) | 1.68 (0) | 1.56 (4) |
| 6:6 bond | 1.37 (1) | 0.55 (1) | 1.70 (1) | 0.73 (1) | 1.00 (2) | 1.92 (0) | 2.01 (2) |
| Corner | 1.24 (1) | 0.75 (1) | 1.19 (3) | 0.86 (1) | 0.91 (4) | 1.56 (0) | 1.49 (4) |
| Hexagon | 1.20 (1) | – | 1.14 (1) | 0.18 (1) | 1.01 (2) | 0.94 (0) | 2.23 (2) |
| Pentagon | 1.38 (1) | – | 1.22 (1) | 0.42 (1) | 0.62 (4) | 1.25 (0) | 1.77 (4) |

Supplementary Table I. **$C_{60}$-$M_1$ binding energies with VASP-GGA.** Binding energies $E_b$ in eV (spin magnetic moment $\mu$ in $\mu_B$) with dipole correction for different metallic species $M_1$ bonded at specific sites of a $C_{60}$ molecule. Nonbonding configurations are indicated with –.

| Species | Valence | $r_s$ ($a_0$) | $r_p$ ($a_0$) | $r_d$ ($a_0$) | $r_f$ ($a_0$) |
|---|---|---|---|---|---|
| Al | $3s^2 3p^1$ | 1.86 | 2.06 | 2.22 | 2.22 |
| Au | $6s^1 5d^{10}$ | 2.29 | 3.14 | 1.20 | 3.14 |
| C | $2s^2 2p^2$ | 1.25 | 1.25 | 1.98 | 1.98 |
| Cu | $4s^1 3d^{10}$ | 2.08 | 2.08 | 2.08 | 2.08 |
| Fe | $4s^1 3p^6 3d^7$ | 2.00 | 2.00 | 2.00 | 1.50 |
| Pd | $5s^1 4d^9$ | 2.58 | 2.71 | 2.45 | 2.45 |
| Ti | $4s^1 3d^3$ | 2.96 | 2.96 | 1.45 | 1.98 |

Supplementary Table II. **Pseudopotentials used for the SIESTA calculations.** Valence configuration and cutoff radii for the $s$, $p$, $d$, and $f$ components (in Bohr) of the pseudopotentials used in the SIESTA calculations. Values for Ti were adapted from E. J. G. Santos, *First principles study of the electronic and magnetic properties of defective carbon nanostructures*, Ph.D. thesis, UPV/EHU, San Sebastian (2011).
